# Human Brucellosis: An Observational Study From a Tertiary Care Centre in North India

**DOI:** 10.7759/cureus.42980

**Published:** 2023-08-05

**Authors:** Renu Kumari, Raj Kumar Kalyan, Asmat Jahan, Amita Jain, Puneet Kumar, K. K Gupta, Anunaya Manoj

**Affiliations:** 1 Department of Microbiology, King George’s Medical University, Lucknow, IND; 2 Department of Microbiology, King George's Medical University, Lucknow, IND; 3 Department of Rheumatology, King George’s Medical University, Lucknow, IND; 4 Department of Medicine, King George's Medical University, Lucknow, IND; 5 Department of Statistics, University of Lucknow, Lucknow, IND

**Keywords:** brucella species, rt-pcr, anti-brucella igg, anti-brucella igm, brucellosis

## Abstract

Aim: The main aim/objective of this study was to detect and characterize the *Brucella *species* *from patients having complaints of joint pain and also to know the potential causes of human brucellosis. In our study, we focused on joint pain symptoms that may be due to arthralgia or arthritis.

Introduction: Brucellosis is a neglected zoonotic disease that affects both humans and animals. In humans, brucellosis begins with chronic illness leading to great financial losses from not being able to work well and continued treatment costs, but few such studies have come from northern India. Joint pain is the common presentation of brucellosis and there are several risk factors associated with brucellosis.

Methods: A total of 200 blood samples were collected from the participants having joints pain from September 2019 to September 2021 at Gandhi Memorial & Associated Hospitals of King George’s Medical University, Lucknow, India, and tested by serology for anti*-Brucella *IgM and IgG enzyme-linked immunosorbent assay (ELISA), molecular* *tests byreverse transcriptase-polymerase chain reaction (RT-PCR),* *conventional polymerase chain reaction (PCR), and automated blood culture system. The anti-*Brucella* IgM and IgG ELISA were performed using the kit from NovaTec Immundiagnostica GmbH (Dietzenbach, Germany). Isolation of DNA was carried out using the QIAamp DNA Mini kit (QIAGEN, Hilden, Germany), and the primers and probes specific for targeted regions (*BCSP31 and IS711 *gene) in the *Brucella *genome were procured from Eurofins Scientific SE (Luxembourg, France), and for internal control from CDC.

Result: The study showed 19 (9.5%) and 23 (11.5%) positive results by anti-*Brucella *IgM ELISA and anti*-Brucella *IgG, respectively, and of these, one (0.5%) was positive for both anti-*Brucella* IgM and anti-*Brucella* IgG ELISA. Out of 19 anti-*Brucella* IgM ELISA positive, eight (4%) samples were positive for PCR/RT-PCR and that was negative for anti-*Brucella* IgG ELISA. All blood culture reports of all patients were negative.

Conclusion: Anti-*Brucella* IgM ELISA was more accurate than anti-*Brucella* IgG ELISA in detecting human brucellosis. Consumption of animal products (i.e. milk, a dairy product of cow, buffalo, goat, and meat of goat) and contact with animals were the main risk factors that were identified for *Brucella* disease.

## Introduction

Brucellosis is the most prevalent and neglected bacterial zoonotic disease, affecting both domestic and wild animal species, besides humans for decades worldwide [1]. In humans, brucellosis begins with chronic illness leading to great financial losses from not being able to work well and continued treatment costs; chronic illness is defined broadly as conditions that last one year or more. With regard to brucellosis, joint pain for more than one year is usually considered as chronic disease [2].

Brucellosis is caused by the Gram-negative, facultative intracellular bacteria belonging to the genus *Brucella* [3]. Of 12 known *Brucella* species, only four species are pathogenic in humans: *Brucella melitensis*, *B. abortus*, *B. suis*, and *B. canis* [4]. These *Brucella* species are resistant to antibiotics because of their surviving capability within phagocytic cells [3,5,6].

Transmission of brucellosis in humans occurs either directly or indirectly through inhalation of an affected animal’s infectious material or consumption of unprocessed animal products [7]. Typically, two to four weeks are required for human brucellosis to develop (starts five days after infection and lasts for six months). Initial brucellosis symptoms in humans include recurrent fever, malaise, bone pain or back pain, tiredness, headache, and night sweats. Signs of human brucellosis include lymphadenopathy, hepatomegaly, and splenomegaly, and it is underdiagnosed worldwide. Lymphadenopathy is misdiagnosed with autoimmune disease, malignancies, drug reactions, etc., hepatomegaly is misdiagnosed with tumors, anemias, heart failure, metabolic disorders, etc., and splenomegaly is misdiagnosed with tuberculosis and endocarditis, etc. [5,7,8].

To identify human brucellosis, several laboratory tests are available including serological and molecular tests like polymerase chain reaction (PCR). Culture is very difficult and takes a long time. PCR is the most accurate test for brucellosis and also it is used as an estimation marker for the course of the disease [9,10]. 

The goal of this investigation was to detect and characterize the *Brucella* species from symptomatic patients having complaints of joint pain and also to know the risk factors. 

This article was previously presented as an abstract at the 45th Annual Conference of the Indian Association of Medical Microbiologists conducted at Kalinga Institute of Medical Sciences (KIMS), Kalinga Institute of Industrial Technology (KIIT) University, Bhubaneshwar, Odisha, India, on November 25, 2022.

The serological and blood culture data used in this article was also published in a previous article by the authors (Kumari et al., 2023) [11].

This article was published as a Preprint on the Access Microbiology server on October 28, 2022 [12].

## Materials and methods

This study included 200 patients aged 7-86 years attending the outpatient and inpatient departments of the Gandhi Memorial & Associated Hospitals of King George’s Medical University, Lucknow, India, with a complaint of joint pain over the period of two years from September 2019 to August 2021. The study was approved by the Institutional Ethics Committee of King George's Medical University, Lucknow, Uttar Pradesh, India (approval number: 1168/Ethics/2019).

We collected 200 blood samples in which the serological and molecular test was performed for all samples. A total of 5 ml of venous blood was collected from each patient in two different vials (a plain vial for serology and an ethylenediamine tetraacetic acid (EDTA) vial for molecular). Data collections of all patients including clinical symptoms, sociodemographic characteristics, and occupations were recorded. Centrifugation, performed for 10 minutes at roughly 1500 rpm, was used to separate the serum from the blood. The serum was aliquoted into two labelled clean cryo vials. One tube was used immediately for serology and another serum vial and EDTA whole blood was immediately frozen at -80 degree freezer till further testing (ELISA and molecular).

Sampling method and sample size

Probability sampling methods and the sample size calculation were made by using the below formula.

n = Z2P (1-P)/d2

n=22 x0.06x94/ (0.5)2 =90.24 for one year

The sample size for one year study is 91 approximately so the sample size for two years will be 200. Where, n= sample size, Z= Z score for a level of confidence (considered 1.96 or 2 at 95% level), P= expected prevalence, and d=precision (considered to be 5%). The seroprevalence of brucellosis is considered to be 6% as per the study of Kaur et al. [13].

Serological assay

The anti-*Brucella* IgM and IgG ELISA were performed as per the manufacturer’s instructions using kits from NovaTec Immundiagnostica GmbH (Dietzenbach, Germany). The calculation was done by following the formula as specified by the manufacturer; for this, absorbance values measured were first converted into NovaTec Units (NTU).

Interpretation of the result was done by following cut-off values, > 11 NTU was considered positive, <9 NTU was considered negative, and 9-11 NTU was considered equivocal as specified by the manufacturer [14,15].

Molecular method

DNA Extraction

DNA extraction was carried out using the QIAamp DNA Mini kit (QIAGEN, Hilden, Germany). A total of 200 µl of whole blood, 20 µl of proteinase K, and 200 µl of lysis buffer were added, and the mixture was incubated at 56^o^C for 10 minutes in a water bath. After 10 minutes, 200 µl of absolute ethanol was put into the lysate. After that, the spin column was cleaned and centrifuged by the manufacturer's instructions. Nucleic acid was eluted with 60 µl of elution buffer in the fresh recovery tube provided in the kit, after five minutes of incubation. For positive control, culture isolates of *B. abortus* and *B. melitensis* were obtained from Indian Veterinary Research Institute, Izatnagar, Bareilly, India. Primers and probes specific for targeted regions (*BCSP31 *and *IS711* gene) in the *Brucella* genome were procured from Eurofins Scientific SE (Luxembourg, France) and for internal control from CDC.

Quantitative Real-Time (qRT)-PCR Assay

The primers and probe employed for the identification of genus *Brucella *were targeted to *Brucella* cell surface protein-31 kDa (BCSP31-genus-specific) genes; those are conserved within the genus *Brucella* and as well as all of its biovars. Target gene Rnase P (RNP) was performed as an internal control for the extraction of *Brucella* (housekeeping gene). Primers as well as fluorescently labeled probes employed in this research are displayed in Table 1. Uniplex RT-PCR tests were executed in a total volume of 10 µl with QuantStudio™ 5 Real-Time PCR System (Thermo Fisher Scientific Inc., Waltham, Massachusetts, United States). PCR reaction 96 well plates contained 8.5 µl Takara uniplex PCR master mixes (Takara Bio Inc., Kusatsu, Shiga, Japan), l.5 µl of primer probes mix of 10 pmol for *Brucella* spp, and 5 µl templates DNA. In the case of RNP, a 10-µl reaction containing 6.25 µl of buffer (Thermo Fisher Scientific Inc.), 1 µl of the primer-probe mix at a concentration of 10 pmol, 0.5 µl of Taq polymerase enzyme, 2.25 µl of H_2_O, and 5 µl of extracted DNA were used. The procedure for the reaction was carried out in Applied Biosystems QuantStudio 5 (0.2ml). The following conditions existed for RT-PCR: an initial denaturation at 95^o^C for 10 minutes, followed by 44 cycles at 95^o^C for 20 seconds, and then at 60^o^C for 90 seconds. Positive and negative controls were used for each experimental study.

**Table 1:** RT-PCR oligonucleotides primers and probes of BCSP31 for Brucella species RT-PCR: real time-polymerase chain reaction

Primer	Sequence (5’‑3’)
BCSP31 Forward primer	GCTCGGTTGCCAATATCAATGC
BCSP31 Reverse primer	GGGTAAAGCGTCGCCAGAAG
RT‑PCR probe	AAATCTTCCACCTTGCCCTTGCCATCA‑FAM/BHQ1
RNAaseP F	CCA AGT GTG AGG GCT GAA AAG
RNAaseP R	TGT TGT GGC TGA TGA ACT ATA AAA GG
RNAaseP PB	CCC CAG TCT CTG TCA GCA CTC CCT TC-FAM-MGBNFQ

PCR Amplification

Table 2 shows primer sequences used for the characterization of *B. abortus* and *B. melitensis* for conventional PCR. Primers were used in a 25 µl reaction that contained 12.5 µl of PCR Master Mix (SuperScript™ III; Thermo Fisher Scientific Inc.), 1 µl of each primer at a concentration of 10 pmol, enzyme 0.5 µl (SuperScript III taq mix; Thermo Fisher Scientific Inc.), DNase free water 5.0 µl, and 5 µl of extracted DNA. Conventional PCR was carried out in a thermal cycler 2720. The cyclic conditions for *B. abortus* and *B. melitensis* were slightly different. For *B. abortus, *it was primary denaturation at 95°C for five minutes followed by 35 cycles of denaturation for the template at 94°C for one minute, 60 seconds of annealing at 61°C, and 60 seconds of extension at 72 °C with a final extension cycle at 72°C for seven minutes. For *B. melitensis*, it was primary denaturation at 95°C for five minutes followed by 36 cycles of denaturation for the template at 94°C for one minute, 60 seconds of annealing at 64°C, and 60 seconds of extension at 72°C with a final extension cycle at 72°C for seven minutes.

**Table 2:** PCR oligonucleotides primers of IS711 for Brucella species PCR: polymerase chain reaction

Primer	Sequence (5’‑3’)	Amplicon size(bp)
IS711, B. abortus Forward primer	TGCCGATCACTTAAGGGCCTTCAT	498 bp
IS711, B. abortus Reverse primer	GAC GAACGGAATTTTTCCAATCCC	
IS711, B. melitensis Forward primer	TGCCGATCACTTAAGGGCCTTCAT	731 bp
IS711, B. melitensis Reverse primer	AAA TCGCGTCCTTGCTGGTCTGA	

*Analysis of the PCR Products* 

PCR products were examined on 2% agarose gel(Thermo Fisher Scientific Inc., Waltham, Massachusetts, United States)in a dilution of 10X TBE Buffer (Tris-borate-EDTA) (1X TBE buffer solution) at room temperature. For gel electrophoresis, 5 µl of the PCR products plus 1 µl 6x loading dye were placed into each gel slot. A ladder of 100 bp (BR Biochem Lifesciences Pvt Ltd, New Delhi, India) was often used to estimate the size of the fragments. The amplified PCR products in agarose gel were viewed and pictured from a gel document system (Alpha-Imager Private Limited, Bengaluru, India). DNase-free water was employed as a negative control, and DNA extracted from *B. abortus* and *B. melitensis* was used as a positive control [16].

Four separate PCR estimations were carried out. The first was for the *Brucella *genus-specific identification, the second PCR for the internal gene or housekeeping gene, and the third and fourth for the species-specific PCR reactions detection of *B. abortus *and *B. melitensis, *respectively.

Blood culture

**Figure 1:**
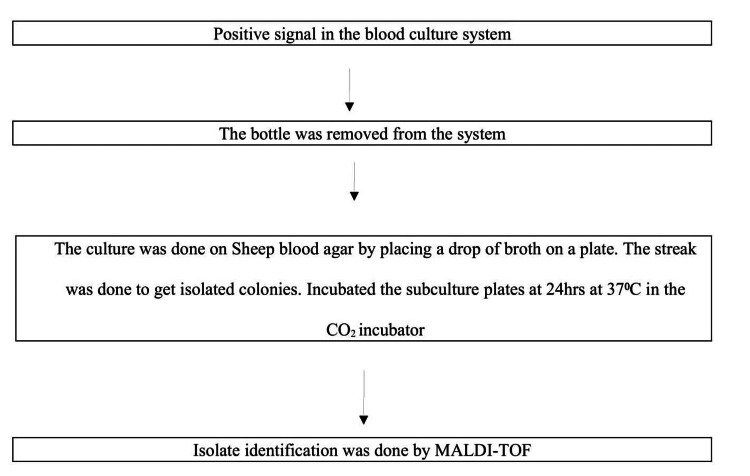
Methodology used for blood culture by automated BacT/ALERT* culture method and identification of isolates. MALDI-TOF: matrix-assisted laser desorption ionization time-of-flight *bioMérieux SA, Marcy-l'Étoile, France Source: Kumari et al., 2023 [11]

For culture, only 130 patients’ blood samples were collected as the remaining patients did not agree to give their samples for culture tests due to them being outdoor patients and the fact that they were not so sick. For culture, 20 ml venous blood from adults and 5-10 ml from children was collected into BacT/ALERT blood culture bottles (bioMérieux SA, Marcy-l'Étoile, France) and subjected to an automated blood culture system (BacT/ALERT) and the cultures were incubated for 21 days at 37°C. Positive BacT/ALERT culture bottles were processed. The methodology is shown in Figure 1.

Statistical analysis

Statistical analysis was done using IBM SPSS Statistics for Windows, Version 21.0 (Released 2012; IBM Corp., Armonk, New York, United States). The prevalence of brucellosis was detected differently depending on the age groups, gender, risk factors, and clinical features. Chi-square test was employed to assess the tests used. Statistical significance was defined as a p-value of <0.05.

## Results

A total of 200 patients presenting with joint pain with or without fever were included in the study. There were 97 (48.5%) males and 103 (51.5%) females. The different age groups included 18-60 years: 161 (82%), older than 60 years: 14 (5.5%), and less than 18 years: 25 (12.5%). The majority of patients were from a rural background (n=129; 64.5%).

Out of the 200 samples, 19 (9.5%) were found positive for anti-*Brucella* IgM and 23 (11.5%) for anti-*Brucella* IgG; of these, 1(0.5%) were positive for both anti-*Brucella* IgM and anti-*Brucella* IgG ELISA. Out of 19 anti-*Brucella* IgM ELISA positive, eight (4%) samples were positive for PCR/RT-PCR and negative for anti-*Brucella* IgG ELISA (Table 3). The sensitivity of anti-*Brucella* IgM ELISA and anti-*Brucella* IgG ELISA was 100%, and 62.5%, respectively, while the specificity was 92.7% for anti-*Brucella* IgM ELISA and 53.1% for anti-*Brucella* IgG ELISA (Table 4). ELISA is the best tool to identify anti-*Brucella* antibodies during an earlier phase of the infection.

**Table 3:** Association between demographic factors and anti-Brucella IgM, anti-Brucella IgG, and RT-PCR/PCR results RT-PCR: real time-polymerase chain reaction

Demographic factors	Anti-*Brucella* IgM N (%)	Chi-square statistic (p-value)	Anti-Brucella IgG, N (%)	Chi-square statistic (p-value)	RT PCR/PCR, N (%)	Chi-square statistic (p-value)
Age						
< =17 years (N = 25)	1 (4.0)	3.152 (0.470)	7 (28.0)	9.324 (0.032)	0 (0.0)	0.743 (0.779)
18-59 years (N = 161)	17 (10.6)		15 (9.3)		8 (5.0)	
>= 60 years (N = 14)	1 (7.1)		1 (7.1)		0 (0.0)	
Sex						
Male (N = 97)	8 (8.2)	1.241 (0.608)	14 (14.4)	6.301 (0.043)	3 (3.1)	0.075 (0.784)
Female (N = 103)	11 (10.7)		9 (8.7)		5 (4.9)	
Residence						
Rural (N = 129)	14 (10.9)	0.914 (0.669)	13 (10.1)	1.226 (0.542)	6 (4.7)	0.066 (0.798)
Urban (N = 71)	5 (7.0)		10 (14.1)		2 (2.8)	
Occupation						
Business (N = 5)	1 (20.0)	7.783 (0.929)	0 (0.0)	15.564 (0.204)	1 (20.0)	5.626 (0.505)
Farmer (N = 10)	1 (10.0)		1 (10.0)		0 (0.0)	
Housewife (N = 73)	10 (13.7)		4 (5.5)		4 (5.5)	
Medical staff (N = 4)	0 (0.0)		0 (0.0)		0 (0.0)	
Student (N = 49)	3 (6.1)		9 (18.4)		1 (2.0)	
Teacher (N = 9)	1 (11.1)		3 (33.3)		0 (0.0)	
Unemployed (N = 29)	2 (6.9)		4 (13.8)		2 (6.9)	
Others (N = 21)	1 (4.8)		2 (9.5)		0 (0.0)	

Table 4 shows the receiver operating characteristic (ROC) analysis for anti-*Brucella* IgM and anti-*Brucella* IgG ELISA, with RT-PCR/PCR as a gold standard.

**Table 4:** Receiver operating characteristic analysis for anti-Brucella IgM and anti-Brucella IgG ELISA ELISA: enzyme-linked immunosorbent assay Source: Kumari et al., 2023 [11]

Characteristics	Anti-Brucella IgM	Anti-Brucella IgG
Sensitivity	100%	62.5%
Specificity	92.7%	53.1%
Area under curve	0.949	0.493

**Figure 2:**
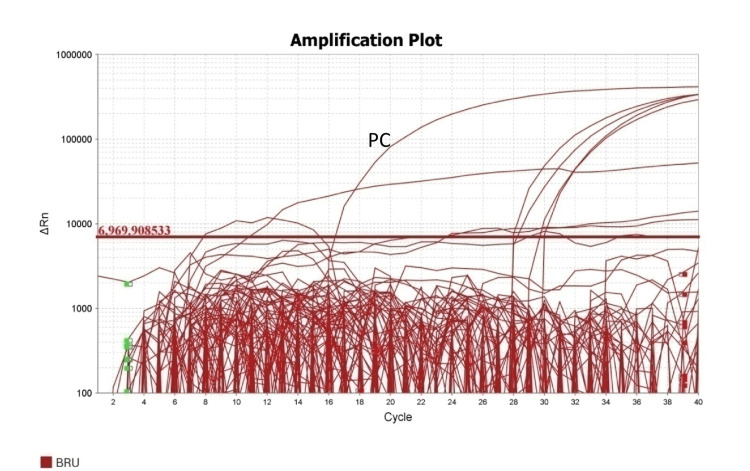
Real-time uniplex Brucella species (Genus specific) PCR amplification plot PCR: polymerase chain reaction; PC: positive control; Δ Rn value: Rn value of experimental reaction − the Rn value of the baseline signal generated by the instrument

**Figure 3:**
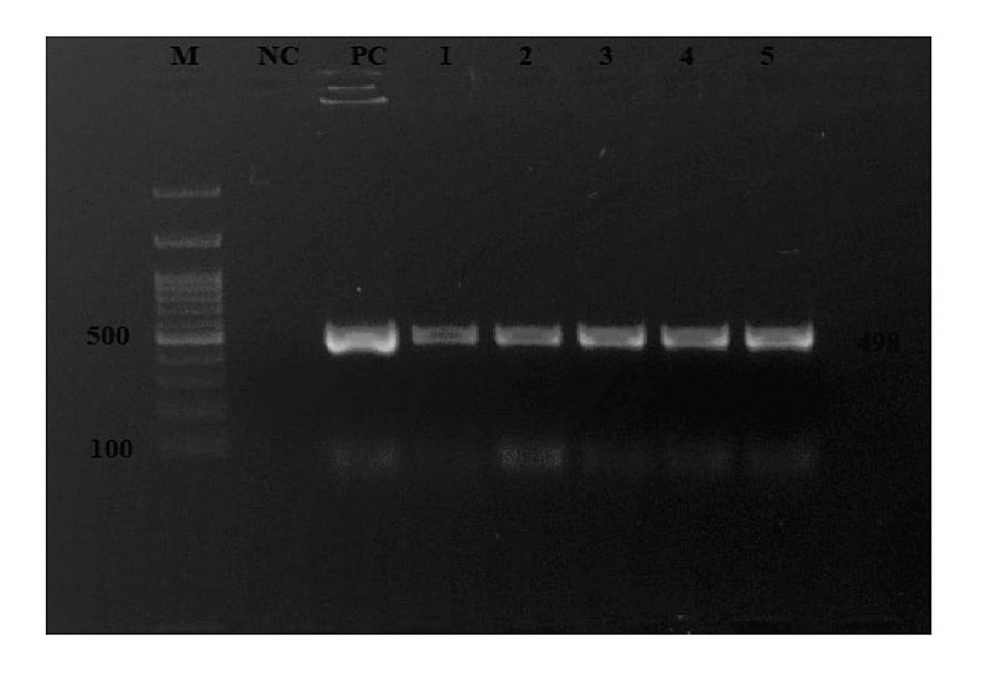
Conventional PCR assay PCR: polymerase chain reaction; M: marker; NC: negative control; PC: positive control Lane 1 to 5 = *Brucella abortus* (498bp) isolate

**Figure 4:**
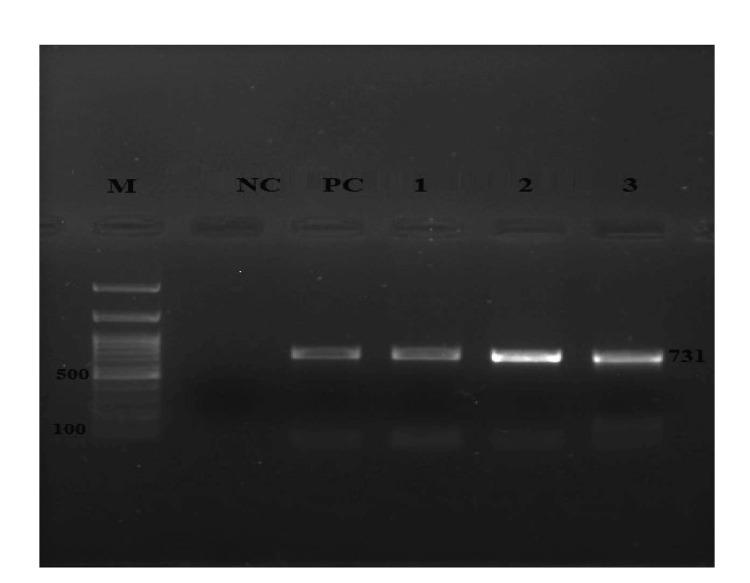
Conventional PCR assay PCR: polymerase chain reaction; M: marker; NC: negative control; PC: positive control Lane 1 to 3 = *Brucella melitensis* (731bp) isolate

Among 200 samples, eight (4%) were positive for brucellosis by using genus-specific BCSP31 RT-PCR (Figure 2) and by using species-speciﬁc gene IS711 conventional PCR. Five samples (2.5%) were found positive for *B. abortus* (Figure 3) and three (1.5%) for *B. melitensis* by PCR (Figure 4).

Analysis showed that brucellosis mostly affects the age group of 7-69 years. The major group affected was housewives (four patients (5.5%) by PCR and anti-*Brucella* IgG, and 10 patients (13.7%) by anti brucella IgM) (Table 3). History of contact with animals (buffaloes, cows, dogs, etc.) was in seven (9.2%) for anti-*Brucella* IgM, eight (10.5%) for anti-*Brucella* IgG, and four (5.3%) for PCR positivity. Consumption of animal products (i.e. milk, a dairy product of cow, buffalo, goat, and meat of goat) was reported in eight (13.8%) for anti-*Brucella* IgM, eight (13.8%) for anti-*Brucella* IgG, and three (5.2%) for PCR (Table 5). The symptoms were fever, headache, chills, and myalgia. All the patients presented with joint pain (n=200; 100%), followed by fever, headache, chills, and myalgia. There was a significant correlation between clinical signs such as night sweats (p = 0.026)) with the positive serology anti-*Brucella* IgG (Table 6). Age (p= 0.032), and sex (p= 0.043) with the positive patients for anti-*Brucella* IgG (Table 3) were both significantly associated with each other. No blood culture was found positive for *Brucella*.

**Table 5:** Association between risk factors and anti-Brucella IgM, anti-Brucella IgG, and RT-PCR/PCR results RT-PCR: real time-polymerase chain reaction

Risk factors	Anti-Brucella IgM, N (%)	Chi-square statistic (p-value)	Anti-Brucella IgG, N (%)	Chi-square statistic (p-value)	RTPCR/PCR, N (%)	Chi-square statistic (p-value)	
Travel history							
Yes (N = 23)	1 (4.3)	0.590 (0.820)	3 (13.0)	0.225 (0.903)	1 (4.3)		0.000 (1.000)
No (N = 177)	18 (10.2)		20 (11.3)		7 (4.0)		
Contact with animal							
Yes (N = 76)	7 (9.2)	2.269 (0.366)	8 (10.5)	0.847 (0.655)	4 (5.3)		0.117 (0.732)
No (N = 124)	12 (9.7)		15 (12.1)		4 (3.2)		
Animal product consumption							
Yes (N = 58)	8 (13.8)	3.035 (0.194)	8 (13.8)	1.261 (0.532)	3 (5.2)		0.020 (0.886)
No (N = 142)	11 (7.7)		15 (10.6)		5 (3.5)		

**Table 6:** Association between clinical features and anti-Brucella IgM, anti-Brucella IgG, and RT-PCR/PCR results RT-PCR: real time-polymerase chain reaction

Clinical features	Anti-Brucella IgM, N (%)	Chi-square statistic (p-value)	Anti-Brucella IgG, N (%)	Chi-square statistic (p-value)	PCR, N (%)	Chi-square statistic (p-value)
Fever						
Yes (N = 84)	5 (6.0)	3.615 (0.163)	11 (13.1)	3.778 (0.151)	2 (5.2)	0.395 (0.530)
No (N = 116)	14 (12.1)		12 (10.3)		6 (2.4)	
Headache						
Yes (N = 58)	7 (12.1)	0.841 (0.763)	3 (5.2)	4.018 (0.133)	5 (8.6)	3.005 (0.083)
No (N = 142)	12 (8.5)		20 (14.1)		3 (2.1)	
Chills						
Yes (N = 38)	5 (13.2)	1.396 (0.406)	3 (7.9)	2.127 (0.360)	2 (5.3)	0.000 (1.000)
No (N = 162)	14 (8.6)		20 (12.3)		6 (3.7)	
Night Sweat						
Yes (N = 12)	0 (0.0)	3.369 (0.216)	0 (0.0)	6.512 (0.026)	1 (8.3)	0.001 (0.976)
No (N = 188)	19 (10.1)		23 (12.2)		7 (3.7)	
Myalgia						
Yes (N = 49)	3 (6.1)	2.237 (0.321)	3 (6.1)	2.219 (0.330)	3 (6.1)	0.205 (0.651)
No (N = 151)	16 (10.6)		20 (13.2)		5 (3.3)	
Arthralgia						
Yes N = 100)	5 (5.0)	5.432 (0.059)	11 (11.0)	0.383 (0.826)	4 (4.0)	0.000 (1.000)
No N = 100)	14 (14.0)		12 (12.0)		4 (4.0)	

## Discussion

Brucellosis is a disease that affects both wild and domestic animals that could be spread among humans by either direct or indirect exposure to infected animals and via unpasteurized milk [17,18]. Signs and symptoms of human brucellosis are not specific. RT-PCR might be the most efficient test for identifying brucellosis brought on by *B. abortus* and *B. melitensis* because of the increased determination limit [19-21].

As a result of the specificity of the chosen genes for detection of *Brucella* in the genus and species levels, the PCR method was appraised as a gold standard technique in the fast and secure diagnosis of brucellosis except the serological testing to determine a more authentic diagnosis of brucellosis notably in cases which are not decisive. Some other studies like that of Kuila et al. correlate with our findings for anti-IgM ELISA and PCR [9]. Similarly, brucellosis prevalence has been widely reported in other parts of India, such as in the Goa region (6.02%), and 4.96% of specimens tested positive by indirect ELISA for anti-Brucella IgM and anti-*Brucella* IgG, respectively, in the study by Pathal et al. [21]. Kaur et al. reported in their study that the prevalence of human brucellosis by ELISA was 6% [13]. In contrast to other studies, Sharma et al. reported overall seroprevalence of brucellosis at 4.96% from Jammu district in North India [22], Barua et al. reported 17.64% *B. melitensis* [23], Rasheduzzaman et al. reported *Brucella* positivity 5.45% by PCR and 3.64% with RT-PCR [24].

Brucellosis is endemic in India [23]. Several studies have reported the prevalence of animal brucellosis in India. Saeed et al. conducted a study in which village-wise variations were found for animal brucellosis in 61.5% of goats, 75% of sheep, 87.5% of buffaloes, and 89% of cattle by Rose Bengal Plate Test (RBPT) and PCR from central Punjab and Pakistan [25]. In 2019, Saeed et al. had reported a prevalence of 35 (3.23%) by RBPT and PCR from Punjab and Pakistan [26]. Another study by Kanani et al. found that in the unorganized sector, 23.70% of sheep (160/675) and 15.99% of goats (283/1769) tested positive for RBPT, whereas 24.44% of sheep (165/675) and 17.24% of goats (305/1769) tested positive for ELISA [27]. Based on the RBPT, the samples revealed that goats had a greater seroprevalence (7.79%, 35/449) than sheep (4.06%, 35/861). Sheep samples had a lower seroprevalence (7.50%, 65/861) than goat samples (9.35%, 42/449) from the organized sector by using I-ELISA from Gujarat [27]. Maansi and Upadhyay reported a 29.61% prevalence in cattle and buffalo in Uttarakhand [28]. In our study, brucellosis was seen to be more frequent in females than males and the most affected were housewives, although it may be due to more symptomatic cases reported in the study.

The limitations of this study were that we were unable to perform the blood culture test on all samples because the patients were not so sick or did not agree to the test. Cross-reactivity was also not ruled out for anti-*Brucella* IgM antibodies. In the future, we will mainly plan or focus on the blood culture and co-infection of brucellosis.

## Conclusions

Human brucellosis is prevalent in Uttar Pradesh, India, while most studies have been done on animal brucellosis. Consumption of animal products and contact with animals were found to be major risk factors for *Brucella* infection. It is important to raise awareness among the general public regarding clinical symptoms, as this will speed up accurate diagnosis. The most important findings in this study on the basis of our objectives are that anti-*Brucella* IgM antibody test was more sensitive for the detection of human brucellosis than anti-Brucella IgG. Joint pain is the common presentation of brucellosis and there are several risk factors associated with human brucellosis. However, there is a paucity of such studies from northern India. Thus, we conducted this study to detect and characterize the *Brucella* species and to know the risk factors associated with human brucellosis.
